# Glibenclamide impairs responses of neutrophils against *Burkholderia pseudomallei* by reduction of intracellular glutathione

**DOI:** 10.1038/srep34794

**Published:** 2016-10-07

**Authors:** Chidchamai Kewcharoenwong, Darawan Rinchai, Arnone Nithichanon, Gregory J. Bancroft, Manabu Ato, Ganjana Lertmemongkolchai

**Affiliations:** 1 The Centre for Research & Development of Medical Diagnostic Laboratories, Faculty of Associated Medical Sciences, Khon Kaen University, Thailand; 2 Department of Immunology and Infection, London School of Hygiene and Tropical Medicine, UK; 3 Department of Immunology, National Institute of Infectious Diseases, Tokyo, Japan

## Abstract

The major risk factor for melioidosis, an infectious disease caused by *B. pseudomallei*, is diabetes mellitus. More than half of diabetic melioidosis patients in Thailand were prescribed glibenclamide. Recent evidence demonstrates that glibenclamide reduces pro-inflammatory cytokine production by polymorphonuclear neutrophils (PMNs) of diabetic individuals in response to this bacterial infection. However, the mechanisms by which glibenclamide affects cytokine production are unknown. We found that PMNs from glibenclamide-treated diabetic individuals infected with live *B. pseudomallei in vitro* showed lower free glutathione (GSH) levels compared with those of healthy individuals. Glibenclamide decreased GSH levels and glutathione peroxidase (GPx) of PMNs after exposed to live *B. pseudomallei*. Moreover, glibenclamide reduced cytokine production and migration capacity of infected PMNs, whereas GSH could restore these functions. Taken together, our data show a link between the effect of glibenclamide on GSH and PMN functions in response to *B. pseudomallei* that may contribute to the susceptibility of diabetic individuals to *B. pseudomallei* infection.

Individual with diabetes are prone to increased susceptibility to infection^1^,^2^. Several infectious diseases are strongly associated with diabetes, including melioidosis^3^. Melioidosis, a serious infection caused by the Gram-negative bacillus *Burkholderia pseudomallei*, is an important clinical problem, which is endemic in South-East Asia and Northern Australia. The distribution of melioidosis in many tropical developing countries is underestimated^4^. It accounts for 20% of cases of community-acquired septicemia in Northeast Thailand, where it is associated with a mortality rate of 50%^3^,^5^ and up to 60% of melioidosis patients have preexisting type 2 diabetes mellitus^6^,^7^.

Animal models have shown that neutrophils play a critical role in protection against *B. pseudomallei* infection^8^,^9^ and these cells are a prominent component of the blood and tissue inflammatory responses in human melioidosis^10^. We have previously explored the mechanism for increased susceptibility to melioidosis in diabetic individuals and found purified PMNs from diabetic individuals to be defective in phagocytosis, migration and apoptosis against *B. pseudomallei*^11^. Moreover, our recent report showed that the sulfonylurea drug, glibenclamide, reduces pro-inflammatory cytokine production by PMNs of diabetic individuals in response to bacterial infection^12^.

Glibenclamide rINN (glyburide USAN) is a broad-spectrum ATP-binding cassette transporter inhibitor^13^ and a K^+^ ATP-channel blocker shown to inhibit cryopyrin/Nalp3 inflammasome activation and proteolytic maturation of IL-1β and IL-18 by caspase-1^14^. This drug therapy is prescribed in around half of diabetics in Northeast Thailand^15^ and widely used in low to middle income settings in other countries. On the other hand, defective IL-12 and therefore IFN-γ production by PBMCs from diabetic individuals which leads to poor control of intracellular bacterial replication is due to a deficiency in intracellular reduced glutathione (GSH) in diabetic PBMCs in response to *B. pseudomallei* and *M. tuberculosis*^16^.

GSH, a ubiquitous sulfhydryl-containing tripeptide produced by most mammalian cells, is usually present as a reduced form, but GSH is converted into its oxidized form (GSSG) by stimulation such as oxidative stress. Therefore, a decreased ratio of reduced to oxidized glutathione is indicative of oxidative stress^17^. Many studies have showed that GSH has protective abilities against the harmful effects of oxidative stress within our body and enhances the functional activity of various immune cells such as natural killer (NK) cells and T cells with an increase in the production of pro-inflammatory cytokines resulting in inhibition in the growth of *M. tuberculosis* inside monocytes and macrophages^16^,^18^. Moreover, inhibition of GSH synthesis in PMNs results in enhanced mycobacterial growth^17^. GSH deficiency may occur in PMNs from diabetic individuals; however, to date, there is no information about the effects of glibenclamide on GSH in PMNs and the consequences of this for antimicrobial resistance.

We hypothesize that glibenclamide may interfere with the intracellular GSH levels of PMNs of diabetic individuals and lead to altered PMN functions such as cytokine production, migration and killing in response to *B. pseudomallei* infection. To address this, we determined intracellular glutathione levels of purified PMNs from diabetic and healthy individuals in response to *B. pseudomallei*. Then, we tested whether exogenous glutathione and N-acetylcysteine (NAC), an aminothiol and synthetic precursor of intracellular cysteine and GSH, could restore immune functions important in resistance to melioidosis.

## Results

### PMNs from diabetic individuals receiving glibenclamide therapy have impaired GSH level

**Figure 1:**
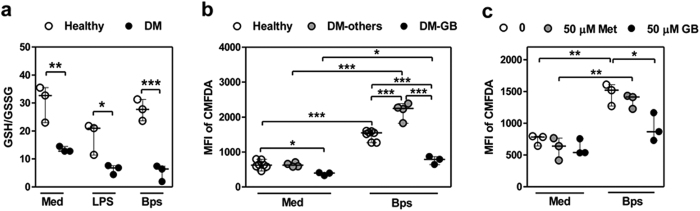
PMNs from diabetic individuals receiving glibenclamide therapy show lower glutathione level. (**a**) Purified PMNs from both healthy (n = 3) and diabetic individuals receiving glibenclamide therapy (n = 3) were co-cultured with *B. pseudomallei* in MOI of 1:1 for 1 h, and then levels of intracellular GSH and GSSG were determined in cell lysates. Data are showed as GSH/GSSG ratio by median with range. Asterisks indicate significant differences of all conditions between healthy and DM individuals by Two Way ANOVA. (**b**) Purified PMNs from both healthy (n = 8), and diabetic individuals receiving other drug therapy (DM-others, n = 4) and diabetic individuals receiving glibenclamide therapy (diabetic-GB, n = 4) were co-cultured with *B. pseudomallei* in MOI of 1:1, and then total glutathione was stained with CMFDA at 18 h for medium (Med) and *B. pseudomallei* (Bps) infection. (**c**) Purified PMNs from healthy individuals (n = 3) were treated by glibenclamide (50 μM) or metformin (50 μM) for 1 h. Drug-treated PMNs were incubated with *B. pseudomallei* (MOI 1:1) for 1 h and then total glutathione was stained with CMFDA at 18 h for medium (Med) and *B. pseudomallei* (Bps) infection and mean fluorescence intensity (MFI) was analyzed by flow cytometry. Statistical analysis was performed using One Way ANOVA. Data are expressed as median with range. ***P < 0.001, **P < 0.01, and *P < 0.05. No asterisk, non significant.

It was previously reported that PBMC of diabetic individuals in Singapore had a deficiency in GSH, which impaired their cytokine response to *B. pseudomallei*^16^. To ask whether changes in GSH was also observed in PMNs, we determined GSH levels in PMNs purified from individuals with very poorly controlled diabetes in the melioidosis endemic region of North East Thailand. These diabetic individuals had all been treated with glibenclamide and were compared against age- and sex-matched healthy individuals. Purified PMNs from the respective groups were incubated in medium alone or in the presence of LPS or live *B. pseudomallei,* and the levels of intracellular GSH and GSSG were detected 1 h later. Unstimulated PMNs from diabetic individuals were found to have a significantly lower GSH/GSSG ratio (Fig. 1a). We next confirmed the total GSH in PMNs by staining with CMFDA (Fig. 1b) and also found that total GSH levels in PMNs from diabetic individuals treated with glibenclamide were significantly lower, while those in PMNs from individuals given other or no treatments (total n = 4) including metformin (n = 1), insulin (n = 2), and no treatment (n = 1) were significantly higher than those of healthy controls. Also *in vitro*, PMNs from healthy individuals pre-treated with glibenclamide but not metformin showed significantly reduced total GSH when compared with untreated cells (Fig. 1c). These data demonstrated that PMNs from diabetic individuals with poor glycemic control receiving glibenclamide therapy were intrinsically impaired in their intracellular GSH levels and this persisted when stimulated with LPS or infected with *B. pseudomallei*.

### Glibenclamide decreases intracellular GSH level of PMNs

**Figure 2:**
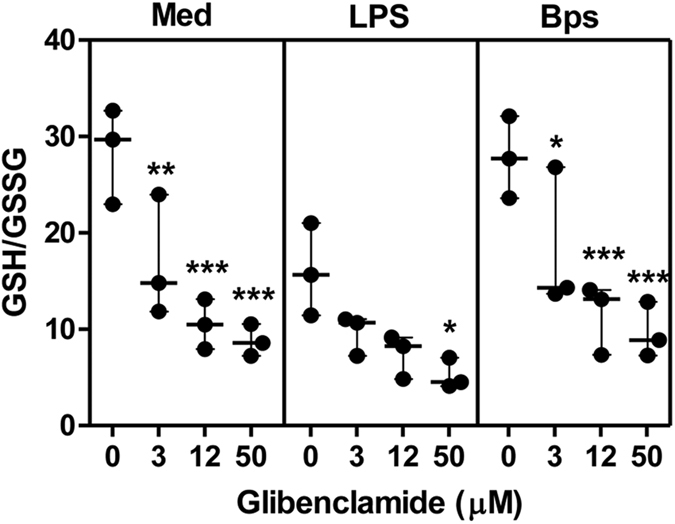
Glibenclamide decreases intracellular glutathione (GSH) level of PMNs from healthy individuals. Purified PMNs from healthy individuals (n = 3) were treated by glibenclamide (GB, 3, 12 and 50 μM) for 1 h. Drug-treated PMNs were incubated with 1 μg/ml LPS or *B. pseudomallei* (MOI 1:1) for 1 h and then the cells were collected for intracellular GSH and GSSG detection. Statistical analysis was performed using Two Way ANOVA comparing each GB treatment group with no GB treatment of each group. The results are expressed as GSH/GSSG ratio by as median with range from 3 independent experiments, and samples were assayed in duplicated. *P < 0.05, **P < 0.01, ***P < *0.001.* No asterisk, non significant.

Since our previous data showed that diabetic individuals who had been treated by glibenclamide had impaired cytokine production^12^, we next determined whether the low GSH levels in *B. pseudomallei*-infected PMNs from diabetes was a result of glibenclamide treatment. Purified PMNs from healthy individuals were pre-treated with glibenclamide before co-culture with either LPS or live *B. pseudomallei*. After incubation for 1 h, PMNs were collected to detect intracellular GSH and GSSG levels. We found that glibenclamide alone significantly reduced GSH/GSSG ratio of PMNs in a dose-dependent manner (Fig. 2). No significant effect of glibenclamide was observed on production of reactive oxygen species (ROS) perhaps due to production of superoxide and hydrogen peroxide depleting GSH^19^,^20^ (Supplementary Fig. S1).

Since it was reported that glutathione peroxidase (GPx) activity was decreased in the cytosol of PMNs from diabetic individuals compared to those from controls^21^, GPx and glutathione reductase (GR) activities were investigated in the same conditions. We found that glibenclamide also significantly reduced GPx activity (Supplementary Fig. S2), but did not affect GR activity (Supplementary Fig. S3). Together, these data demonstrated that glibenclamide reduced intracellular GSH level and GPx activity but not ROS production and GR activity of PMNs in response to LPS stimulation or *B. pseudomallei* infection.

### *In vitro* addition of GSH improves IL-8 production of PMNs from diabetic individuals receiving glibenclamide therapy

**Figure 3:**
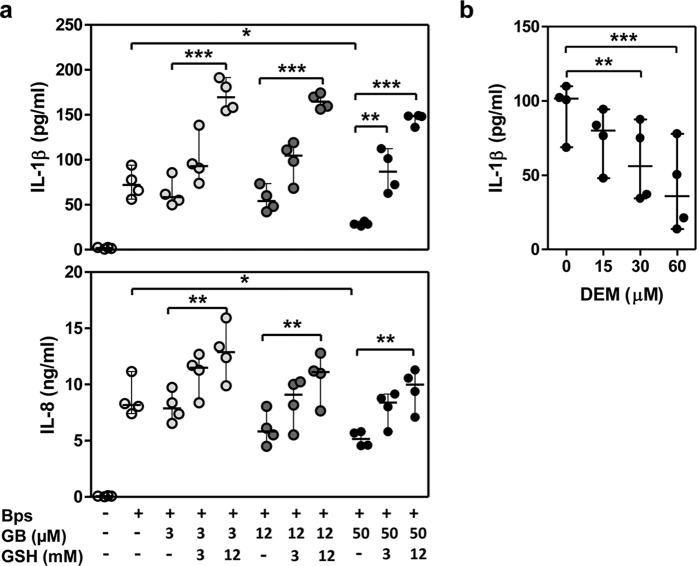
Glutathione enhances cytokine production of glibenclamide-treated PMNs. (**a**) Purified PMNs from healthy individuals were treated by glibenclamide (GB, 3, 12 and 50 μM) and glutathione (GSH, 3 and 12 mM) for 1 h. After treatment PMNs were infected with *B. pseudomallei* (MOI 0.3:1) for 18 h, the supernatants were collected. Statistical analysis was performed using One Way ANOVA comparing each GSH treatment group with no GSH of each GB treatment group and paired *t* test comparing each GB alone group with Bps alone. (**b**) Purified PMNs were treated by diethyl maleate (DEM, 15, 30 and 60 μM) for 1 h. Treated PMNs were infected with *B. pseudomallei* (MOI 0.3) and the supernatant was collected at 18 h. Statistical analysis was performed using One Way ANOVA comparing each DEM treatment group with no DEM treatment group (0). The results are expressed as median with range, and samples were assayed in duplicate from 4 independent experiments. *P < 0.05, **P < 0.01, ***P < *0.001.*

Cytokine production by neutrophils is an important component of innate immunity which is reduced in diabetic individuals^12^. To ask whether exogenous addition of GSH could restore the impaired cytokine response of glibenclamide-treated PMNs, purified PMNs from healthy individuals were pre-treated with glibenclamide alone over a range of 3, 12 and 50 μM or combined with GSH and then infected with *B. pseudomallei* for 18 h. PMNs treated with glibenclamide alone showed a significant, dose dependent reduction IL-1β and IL-8 levels in response to *B. pseudomallei* infection *in vitro*, in agreement with what has been previously reported^12^ whereas GSH significantly enhanced the production of both cytokines (Fig. 3a). *Moreover,* our data showed that GSH depletion by diethylmaleate (DEM) treatment reduced IL-1β production of PMNs in response to *B. pseudomallei (*Fig. 3b). Under these conditions, addition of GSH alone or DEM alone, in the absence of bacteria, did not induce detectable cytokine production (data not shown). These data suggested that exogenous GSH could restore the impaired cytokine responses of glibenclamide-treated PMNs.

**Figure 4:**
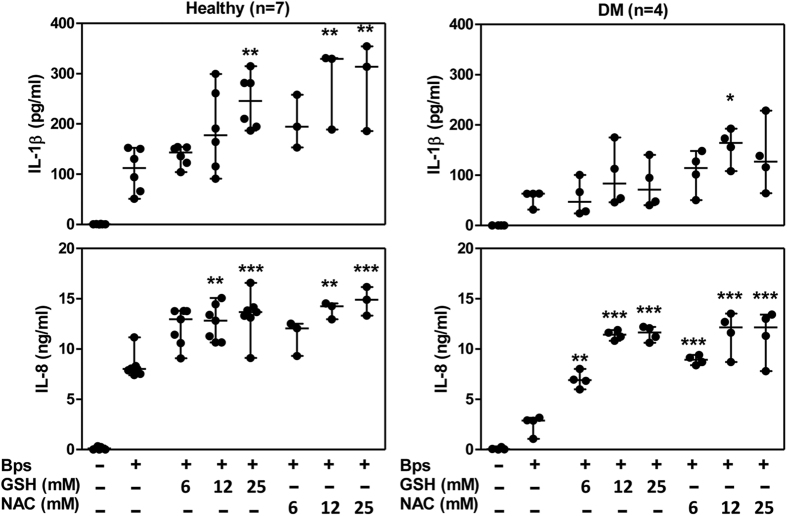
Glutathione and N-acetylcysteine enhances IL-8 production of PMNs from diabetic individuals receiving glibenclamide therapy. The concentrations of IL-8 and IL-1β were determined by ELISA. PMNs from healthy (n = 7) and diabetic patients (n = 4) were pre-treated by glutathione (GSH) or N-acetylcysteine (NAC) at varied concentrations (6.25, 12.5, 25 and 50 mM) and then infected with *B. pseudomallei* in MOI of 0.3:1. The supernatants were collected at 18 h. Asterisks indicate significant differences between non-treated and treated conditions by One Way ANOVA and expressed as median with range, and samples were assayed in duplicate. ***P < 0.001, **P < 0.01, and *P < 0.05. No asterisk or ns, non significant. ND, not determine.

The above observations led us to determine the effect of N-acetylcysteine (NAC), an aminothiol and synthetic precursor of intracellular cysteine and GSH, on cytokine production of PMNs from diabetic individuals who had been treated by glibenclamide. NAC, a prodrug that supplies bioavailable cysteine necessary for the replenishment of GSH, is routinely administered clinically to overcome pharmacologically induced GSH deficiency^22^,^23^. Either GSH or NAC was added exogenously to the cells and the cytokine levels were monitored after *B. pseudomallei* infection. NAC significantly enhanced both IL-1β and IL-8 production by PMNs from both healthy and diabetic individuals in a dose-dependent manner. However, there was a trend for IL-1β production by cells of diabetic individuals to be increased by GSH but this was not statistically significant (Fig. 4). Treatment with GSH or NAC alone has no effect. However the magnitude of IL-1β production of PMNs from diabetic individuals was still low. Together, these findings indicated that both GSH and NAC could improve cytokine production, especially IL-8, of PMNs from diabetic individuals in response to *B. pseudomallei* infection.

### Glutathione improves impaired migration activity of PMNs from glibenclamide-treated diabetic individuals

**Figure 5:**
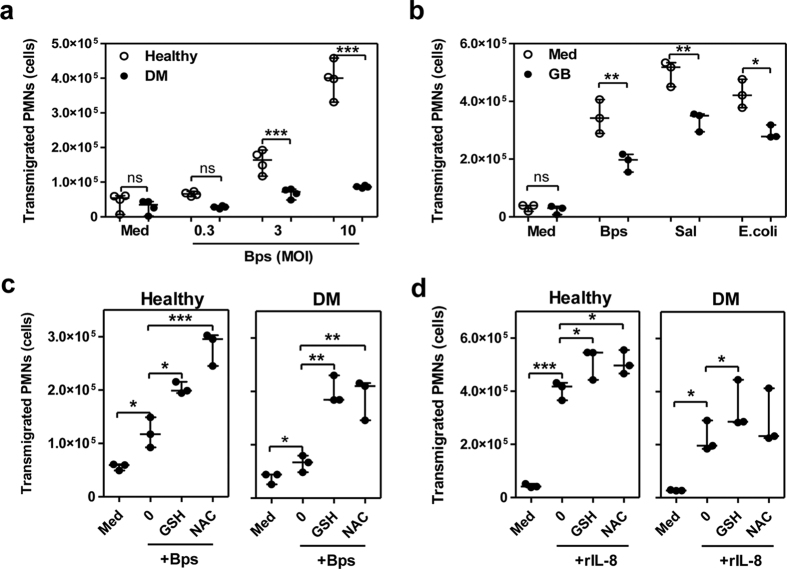
Glutathione and N-acetylcysteine improves impaired migration activity of PMNs. (**a**) Numbers of transmigrating PMNs of diabetic (n = 4) comparing with healthy (n = 4) individuals. Purified PMNs were added into the upper wells and co-cultured with live *B. pseudomallei* put into the lower wells. After co-culture for 1 h, transmigrating PMNs were counted by flow cytometry. Asterisks indicate significant differences between healthy and DM individuals at the same concentrations. (**b**) Purified PMNs from healthy individuals (n = 3) were treated by 50 μM glibenclamide (GB) before infection with *B. pseudomallei, S. enterica serovar Typhimurium (Sal)*, and *E. coli* at MOI of 10:1. Asterisks indicate significant differences between glibenclamide treatment and medium alone in the same conditions by paired *t* test. (**c**) Purified PMNs from healthy (n = 3) and diabetic (n = 3) individuals were treated by 12.5 mM glutathione or 12.5 mM NAC before addition to the upper wells and co-cultured with *B. pseudomallei* at MOI of 3:1 or (**d**) with 40 ng/ml of recombinant human IL-8 (rIL-8). Asterisks indicate significant differences between medium control and not-treated (0) or between not-treated and other conditions. *P* values were calculated by using One Way ANOVA. Data are expressed as median with range, and samples were assayed in duplicate. No asterisk or ns; non significant, *P < 0.05, **P < *0.01.*

Since glibenclamide reduced IL-8 production, which plays a role in the induction of PMN infiltration, purified PMNs from both diabetic and healthy individuals were investigated for transmigration in response to *B. pseudomallei* infection over a range of 0.3, 3 and 10 MOIs. We found that the number of transmigrated PMNs to *B. pseudomallei* of diabetic individuals who had been treated with glibenclamide was significantly lower than those of healthy controls (Fig. 5a). Further, glibenclamide-treated PMNs from healthy individuals also showed significantly decreased transmigration when compared with non-treated cells (Fig. 5b) in response not only to *B. pseudomallei but also other bacteria; S. enterica serovar Typhimurium and E. coli*. To test the effect of GSH on the migration capacity of PMNs, purified PMNs from diabetic and healthy individuals were supplemented with GSH or NAC prior to exposure to *B. pseudomallei or recombinant IL-8.* Diabetic PMNs treated with GSH and NAC showed significant improvement in their migration responses to bacteria and recombinant IL-8 (Fig. 5c,d). Together, these data suggested that glibenclamide impaired PMN migration of diabetic individuals while GSH and NAC restored this function.

### *In vitro* addition of glutathione affects phagocytosis and killing of PMNs from diabetic individuals receiving glibenclamide therapy

**Figure 6:**
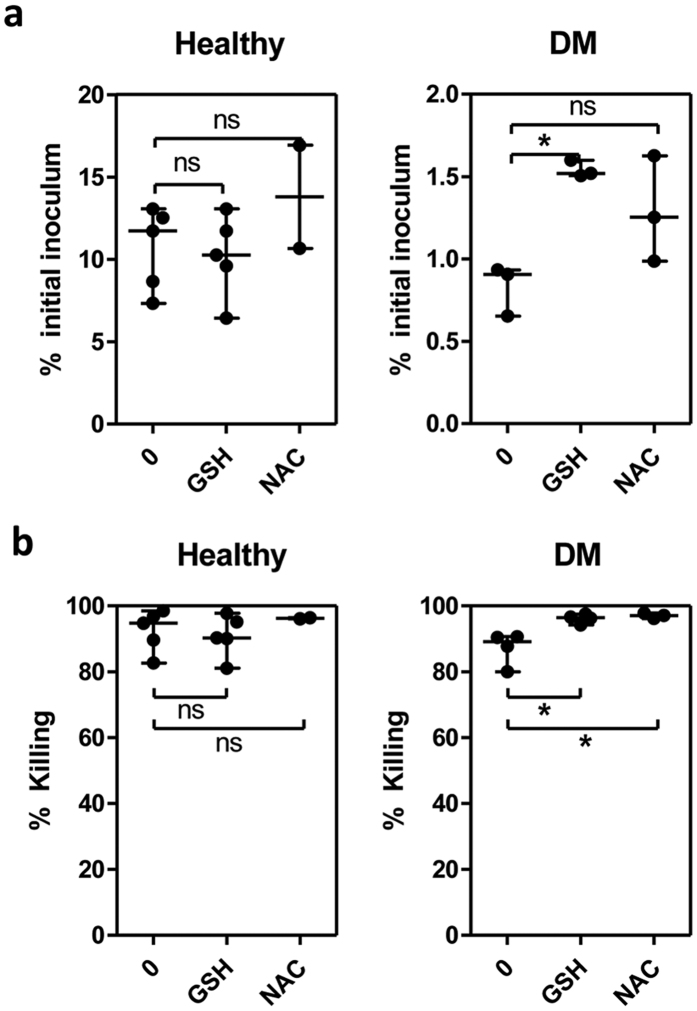
Glutathione and N-acetylcysteine affects bacteria uptake and bactericidal activity of PMNs from diabetic individuals. (**a**) Purified PMNs from healthy (n = 5) and diabetic (n = 4) individuals were treated by 12.5 mM glutathione or 12.5 mM NAC before co-culture with live *B. pseudomallei* at an MOI of 1:1 for 30 min, and extracellular organisms were killed by incubation with 250 μg/ml kanamycin for another 30 min before the PMNs were lysed for bacterial counting (initial inoculum time) and after 3 h. Intracellular bacteria were quantified by colony plating at the indicated time points, and the results are expressed as percentages of the initial inocula for each individual, which were calculated by dividing the number of recovered bacteria by the total number of *B. pseudomallei* cells added. (**b**) %Killing was calculated by dividing the decreased number of bacteria at 3 h by the number of bacteria at initial inoculum time. Asterisks indicate significant differences and *P* values were calculated by using One Way ANOVA. Data are expressed as median with range, and samples were assayed in duplicate. No asterisk or ns; non significant, *P < 0.05, **P < *0.01*.

To further examine the effect of GSH and NAC on the antimicrobial functions of impaired PMNs, purified PMNs from either healthy individuals or diabetics with poor glycemic control were exposed to live *B. pseudomallei in vitro* and the extent of phagocytosis and killing were determined by counting intracellular bacteria. The percentage of initial inoculum, which measures phagocytic activity, showed that GSH supplementation partially increased phagocytosis of the bacteria by PMNs (Fig. 6a) and both GSH and NAC induced PMN mediated killing of *B. pseudomallei* (Fig. 6b) in diabetic individuals, while supplementation did not affect PMNs from healthy controls. However, the supplementation by GSH and NAC could only show limited improvement of phagocytosis and killing and could not restore these functions of PMNs from diabetic individuals who had been treated by glibenclamide to that of healthy controls.

## Discussion

Diabetic individuals commonly show increased susceptibility to bacterial infections^24^,^25^,^26^. In Thailand, type 2 diabetes mellitus is the most common underlying condition associated with melioidosis. Two main possible mechanisms have been proposed to explain the high susceptibility of diabetic individuals under hyperglycemic conditions in response to *B. pseudomallei* infection. First, diabetic individuals in Singapore had a defective IL-12 and therefore IFN-γ production by PBMCs leading to poor control of intracellular bacterial replication and the impairment response to a deficiency in intracellular reduced glutathione (GSH) in diabetic PBMCs in response to *B. pseudomallei*^16^. Second, we previously demonstrated that polymorphonuclear neutrophils (PMNs) from diabetic individuals had impaired functions, including phagocytosis, migration, and apoptosis, encountered to *B. pseudomallei* infection^11^. We proposed this is at least in part due to a pattern of glibenclamide-induced immunosuppression, wherein IL-1β and IL-8, proinflammatory cytokines, belonging to PMNs were significantly decreased in diabetic individuals who have been treated by either glibenclamide alone or combination with metformin^12^.

More than half of all diabetic melioidosis patients in Thailand were prescribed glibenclamide, and those on this oral hypoglycemic agent had a survival benefit compared with nondiabetic melioidosis patients and with diabetic melioidosis patients on other diabetic medications, such as metformin and insulin^15^,^20^. These patients are suggested to be less likely to undergo severe hyper-inflammatory responses in septic melioidosis, which may be explained, at least in part, by our data suggesting that glibenclamide could decrease GSH and IL-1β production of PMNs. These might be due to the reduction of GSH linked to the effect of glibenclamide during melioidosis. Nevertheless, glibenclamide treatment with poor glycemic control may place diabetic patients at a disadvantage for onset or a recurrence of *B. pseudomallei* infection. Glibenclamide is a widely used antidiabetic drug not only in Thailand but also in other low- and middle-income countries^27^ but notably not in Singapore. It is still used with elderly diabetic individuals in these countries, though the WHO model list of essential medicines (2015) reported that it is not suitable for those aged above 60 years^28^. The use of glibenclamide is likely linked to poverty and poor economic conditions, whereas some upper-income countries use other drugs of choice. The effect of glibenclamide was attributed to its anti-inflammatory nature, as it had been shown to be able to inhibit NLRP3 inflammasome activation^20^. However, there is no evidence on GSH deficiency and its potential effects on may occur in PMN and whether this is linked to glibenclamide. Thus, our present study aimed to determine the effect of glibenclamide on the intracellular GSH level from PMNs of diabetic individuals and the impact of this on PMN function.

Our findings indicate that diabetic individuals who had been treated by glibenclamide show impaired cytokine production and migration activity of PMNs in response to *B. pseudomallei* by reduction of intracellular GSH levels and GPx activity. However, in streptozotocin-induced diabetic rat models, decreased levels of GSH in the brain, muscle, pancreatic β-cells and blood have been reported. In addition, the activity of antioxidant enzymes such as GPx, among others, has been found to be down-regulated^29^,^30^,^31^,^32^. Also we found that PMNs from healthy controls incubated with glibenclamide reduced intracellular GSH so the reduction of GSH may concern both poor glycemic control and drug treatment in diabetic individuals. Not only our data in PMNs, but a recent study in PBMCs obtained from poor glycemic control diabetes also showed low intracellular GSH levels are critical in allowing better initiation of the intracellular infection and provide an explanation for the increased susceptibility of diabetic individuals to melioidosis. The low intracellular GSH/GSSG ratio could also affect disease progression and outcome^16^. Our data also showed that depletion of GSH in PMNs supported the conclusion that GSH deficiency contributes to decreased IL-1β production.

In contrast, we found that PMNs from diabetic individuals with poor glycemic control but treated by insulin had increased GSH in response to *B. pseudomallei* compared with healthy or glibenclamide-treated diabetic individuals. This result was consistent with a previous study in streptozocin (STZ)-induced diabetic rats shown that Insulin restored hepatic glutathione concentrations to normal levels^33^. The data implies that reduction of intracellular GSH in PMNs is mainly caused by glibenclamide treatment.

Another study in mouse models of sepsis has reported that migration of PMNs to a site of infection (the skin) and to a distant site (the lung) is differently regulated, and optimal GSH levels are important for an efficient response to sepsis^34^. We found that normal GSH status is essential for proper PMN migration in response to live bacteria, in that GSH impairment with glibenclamide decreases PMN transmigration after co-cultured with *B. pseudomallei*. Small but statistically significant changes in PMN numbers have been implicated in altered susceptibility to infection with other pathogens; for example, in the context of *M. tuberculosis*, risk of TB infection was inversely and independently associated with peripheral blood neutrophil count in contacts of patients diagnosed with pulmonary TB^35^. Moreover it has been demonstrated in *Ifnar1*^−/−^ mice infected with *M. tuberculosis* that a small reduction of PMN recruitment to the site of infection also showed a significant decrease in survival rate^36^. Studies in humans and mice have identified PMNs and PMN-recruiting chemokines at sites of *B. pseudomallei* infection, particularly in association with abscess formation^8^,^37^. Clinical susceptibility and the development of severe bacteremic disease are often associated with diabetes mellitus^6^, a condition in which PMN migration is known to be impaired. This small difference of PMN numbers migrating to the infected site, together with impairment of other cell functions, might lead to increasing susceptibility to bacterial infection.

This is an important distinction, since the role of glibenclamide in intracellular GSH is uncertain. We believe that changes in GSH levels due to glibenclamide, represent a novel and potentially important effect of the diabetic drug on innate defense mechanism adopted by human PMNs to control *B. pseudomallei* infection through GSH redox. The explanation of GSH reduction by glibenclamide treatment may be due to the following possible mechanisms: 1) we found that glibenclamide did not affect the regenerated GSH cycle by the enzyme GR which performs the reduction of GSSG to GSH, and oxidation of NADPH to NAD^+^^17^, but it reduced GPx levels which performs the reduction of hydrogen peroxide to water, while linking two GSH molecules together by a disulfide bridge to form GSSG. 2) Glutathione-S-transferase omega 1 (GSTO1) is a recently characterized cysteine containing enzyme with the capability to bind and release GSH *in vitro*^38^. GSTO1 was found to associate with ASC, a component of the inflammasome, suggesting that it might play a role in NLRP3 and AIM2 inflammasome signalling and could indeed be a target of diarylsulfonylurea containing compounds called Cytokine Release Inhibitory Drugs (CRIDs) that inhibited the post-translational processing of IL-1β^39^. Thus, glibenclamide might affect these mechanisms and cause reduced intracellular GSH, which in turn leads to decrease bactericidal activity of PMNs from diabetic individuals.

Moreover, we experimented with exogenous GSH and N-acetylcysteine (NAC) and whether they could restore immune functions. We found that the addition of GSH or NAC significantly boosted both cytokine production and migratory functions of PMNs from diabetic individuals who had been treated by glibenclamide. However, GSH restored the magnitude of IL-1β less than IL-8, perhaps because of pre-existing impairment of IL-1β in PMNs of poor glycemic control diabetic individuals^12^. Either GSH or NAC also enhanced the direct antimicrobial functions of PMNs from diabetic individuals who had been treated with glibenclamide or in combination with metformin. Differently from cytokine production and recruitment, GSH could not fully restore the phagocytic activity of PMNs from diabetic individuals to those from healthy controls. It might imply that GSH had only a partial contribution to the altered phagocytic activity of PMNs from diabetic individuals which may be also affected by preexisting defects of poor glycemic control^11^ through oxygen uptake^40^ and membrane synthesis^41^. In contrast, both GSH and NAC increased the killing activity of diabetic PMN.

In conclusion, we have shown that glibenclamide reduced intracellular GSH concentrations in PMNs from diabetic individuals with poor glycemic control, resulting in a significant decrease in IL-1β and IL-8 production of PMNs in response to *B. pseudomallei infection.* This in turn impairs the migratory capacity of PMN from diabetic individuals as compared with healthy controls. Our work has therefore revealed a new link between the effect of glibenclamide on GSH and PMN functions in response to *B. pseudomallei* that may affect host susceptibility to *B. pseudomallei* infection in diabetic individuals. However, there is clearly a delicate balance between host defense and inflammation. GSH reduction, which is often associated with sepsis^42^, might be detrimental by decreasing PMN recruitment and so increasing the risk of infection but conversely reduce the extent of PMN mediated organ damage. We are not aware of current pharmacological approaches which can selectively inhibit the pathogenic aspects of inflammation without potentially interfering with the benefits of phagocyte recruitment and activation. One multicenter trial found that direct IL-1 blockade does not improve survival in all-cause sepsis^43^. It is unclear whether any benefit would be seen were glibenclamide started following the onset of infection in nondiabetic patients. However, the central role of GSH in both monocyte^16^ and PMNL responses to *B. pseudomallei* strengthens the need to consider whether adjunctive treatment with thiol antioxidants and GSH-repleting agents might be of benefit in individuals from low income countries where glibenclamide is the only option for management of Type II diabetes. A previous study showed that oral NAC supplementation in diabetic individuals is only sufficient to increase intracellular GSH content in blood cells but not sufficient to improve bactericidal activity in monocytes^44^. However, their study showed the complication of only poor glycemic control in diabetic patients and excluded diabetic individuals who had glibenclamide treatment. These agents might help reorient cytokine production and migration activity of PMNs from glibenclamide treated individuals in a way that is more favorable to the host. In a mouse model, NAC enhanced PMN infiltration in the site of infection improved the survival outcome^34^. This strategy could be combined with supplementation with glutamine, which seems to play an important role in PMN functions^45^. Given the relatively low costs of these treatments, they may be considered as preventive supplements in diabetics with poor glycemic control at high risk of exposure *B. pseudomallei* in endemic areas.

## Methods

### Blood samples

We enrolled 24 diabetic and 31 healthy Thai individuals. All individuals were defined here as aged over 35 years and matched by sex and age^11^. None of the individuals had any signs of acute infectious disease in the three months prior to sampling. The healthy control group consisted of individuals with normal fasting blood glucose levels (3.9–6.2 mmol/L) and normal glycosylated hemoglobin A1c (HbA1c) levels (<6.5%). HbA1c was detected by immunoturbidimetric assay and the results were reported following the National Glycohemoglobin Standardization Program (NGSP). The healthy individuals were recruited from both blood bank donors and healthy university staff, who live in the same endemic area as diabetic individuals. The diabetic individuals involved in this study had all been treated with glibenclamide either alone or in combination with metformin with very poor glycosylated hemoglobin A1c (HbA1c) levels (>8.5%)^11^, unless stated otherwise. All diabetic individuals were treated at the Outpatient Department, Khon Kaen Hospital and Khon Kaen Primary Medical Service. The exclusion criteria for both healthy and diabetic individuals were impaired renal function which causes secondary immunosuppression and is itself a separate risk factor or melioidosis, defined by a serum creatinine level of ≥0.11 mmol/L. The sample collection and analysis of all samples was approved by the Khon Kaen University Ethics Committee for Human Research and the Khon Kaen Hospital Ethics Committee (HE470506). The study was carried out in accordance with the approved guidelines and all subjects provided written informed consent. Each experiment was performed with both healthy and matching DM subjects, generally one healthy versus one matching DM subject, unless stated otherwise.

### Microorganisms

*B. pseudomallei* K96243 strain was grown to mid-logarithmic phase at 37 °C in Luria-Bertani (LB) broth. Bacterial growth was assessed by measuring the optical density at 600 nm. Generally, an absorbance index of 1 was equivalent to10^9^ CFU/mL of bacteria and the number of viable bacteria (colony-forming units) in inocula was determined by retrospective plating of serial ten-fold dilutions on LB agar. Live *B. pseudomallei* was handled under the US Centers for Disease Control regulations for research at biosafety containment level 3. *Salmonella enterica* serovar Typhimurium ATCC 13311 and *Escherichia coli* ATCC 25422 were obtained from the Microbial Culture Collection Center, Thailand, grown overnight in LB broth at 37 °C and enumerated as above.

### Isolation of PMNs

We isolated PMNs from heparinized venous blood by dextran sedimentation and Ficoll-Paque centrifugation, as previously described^11^, and the resulting cell preparation was confirmed to consist of >95% PMNs by flow cytometry and by CD14 staining. The morphology of PMNs was also determined by Giemsa staining and microscopy, and the cell viability was >98%, as determined by trypan blue exclusion.

### Detection of intracellular GSH and GSSG concentration by colorimetric assay

In some experiments, purified PMNs at a concentration of 1 × 10^6^ cells in RPMI 1640 were pre-treated for 1 h with varied concentrations of glibenclamide (Sigma) which are comparable to human blood concentrations achieved following a standard 20 mg oral dose^46^ before co-culture at 37 °C, 5% CO_2_ for 1 h with live *B. pseudomallei* at a multiplicity of infection (MOI) of 1:1 or with 1 μg/ml of LPS (from *Escherichia coli*, Sigma). Intracellular GSH and oxidative glutathione (GSSG) concentrations in PMNs were determined using a Glutathione Colorimetric Detection Kit (Arbor Assays) according to the manufacturer’s instructions.

### Determination of intracellular GSH level by 5-chloromethylfluorescein-diacetate (CMFDA)

Purified PMNs at a concentration of 2.5 × 10^6^ cells/ml in RPMI 1640 (Gibco) were incubated at 37 °C, 5% CO_2_ for 18 h with live *B. pseudomallei* at a multiplicity of infection (MOI) of 1:1. In some experiments, the following reagents were added to PMNs for 1 h prior to infection. The reagents were 50 μM glibenclamide in DMSO (Sigma), and 50 μM metformin in water for injection (Sigma). Then, cells were stained by 0.5 μM CMFDA (Invitrogen) to detect total GSH (specificity 95%)^47^ according to the manufacturer’s instructions and mean fluorescence intensity (MFI) was analyzed by flow cytometry (FACSCalibur; BD Biosciences) for 10,000 events gated on the granulocyte population.

### Measurement of cytokine production

Unless stated otherwise, purified PMNs at a concentration of 2.5 × 10^6^ cells/ml in RPMI 1640 culture medium (Gibco) with 10% fetal bovine serum were incubated at 37 °C, 5% CO_2_ for 18 h with *B. pseudomallei* at a multiplicity of infection (MOI) of 0.3:1. In some experiments, the following reagents were added to PMNs for 1 h prior to infection. The reagents were varied concentrations of glibenclamide in DMSO (Sigma), diethyl maleate (DEM) in ethanol (Sigma) and both GSH (Sigma) and N-acetylcysteine (NAC) in water for injection (Sigma). The supernatants were stored at −80 °C until the cytokine assay. When indicated, we assessed IL-1β and IL-8 concentration in the supernatant of PMN cultures in duplicate by ELISA (BD Biosciences) according to the manufacturer’s instructions.

### Determination of PMN migration

Purified PMNs at a concentration of 5 × 10^6^ cells/ml were incubated in the upper chamber of 3-μm-pore-size Transwell plates (Corning Life Sciences), *B. pseudomallei* at an MOI of 0.3:1, 3:1, or 10:1 was placed in the lower 0.5 ml chamber, and the plates were incubated at 37 °C for 1 h. Transmigrated PMNs in the lower chamber with reference beads were counted by flow cytometry (FACSCalibur; BD Biosciences) for 10,000 events gated on the granulocyte population. The absolute count of transmigrated PMNs was calculated by relating the number of cells counted to the total number of bead events. In some experiments, purified PMNs were pre-treated with 50 μM glibenclamide (Sigma), 12.5 mM GSH (Sigma) and 25 mM NAC (Sigma) at 37 °C for 1 h prior to the test for migration.

### Determination of PMN mediated killing of *B. pseudomallei*

Purified PMNs were treated by 12.5 mM glutathione or 12.5 mM NAC before co-cultured with live *B. pseudomallei* at an MOI of 1:1 for 30 min, and extracellular organisms were killed by incubation with 250 μg/ml kanamycin for another 30 min before the cells were lysed for bacterial counting (initial inoculum time) and after 3 h. Intracellular bacteria were quantified by colony plating at the indicated time points, and the results are expressed as percentages of the initial inocula for individuals, which were calculated by dividing the number of recovered bacteria by the total number of *B. pseudomallei* cells added. (B) %Killing was calculated by dividing the decreased number of bacteria at 3 h by the number of bacteria at initial inoculum time.

### Statistics

Statistical analysis (One Way and Two Way ANOVA with post-test Bonferroni) was performed by using Graphpad PRISM statistical software version 5 (Graphpad 5). P values ≤ 0.05 were taken to be significant. The power of each test was calculated by post-hoc power analysis with 95% confidence interval and more than 80% was acceptable for all experiments.

## Additional Information

**How to cite this article**: Kewcharoenwong, C. *et al*. Glibenclamide impairs responses of neutrophils against *Burkholderia pseudomallei* by reduction of intracellular glutathione. *Sci. Rep.* **6**, 34794; doi: 10.1038/srep34794 (2016).

## Supplementary Material

Supplementary Information
