# Supplementary material for: Glibenclamide impairs responses of neutrophils against Burkholderia pseudomallei by reduction of intracellular glutathione
Source: Sci Rep. 2016 Oct 7;6:34794. doi: 10.1038/srep34794 (PMC5054430; doi:10.1038/srep34794)
Supplement: Supplementary Information [file srep34794-s1.pdf]

Supplementary information

Glibenclamide impairs responses of neutrophils against *Burkholderia pseudomallei* by reduction of intracellular glutathione

Chidchamai Kewcharoenwong[1], Darawan Rinchai[1], Arnone Nithichanon[1], Gregory J. Bancroft[2], Manabu Ato[3]* and Ganjana Lertmemongkolchai[1]*

[1]The Centre for Research & Development of Medical Diagnostic Laboratories, Faculty of Associated Medical Sciences, Khon Kaen University, Thailand, [2]Department of Immunology and Infection, London School of Hygiene and Tropical Medicine, UK, and [3]Department of Immunology, National Institute of Infectious Diseases, Tokyo, Japan.

Supplementary Figures S1-S3

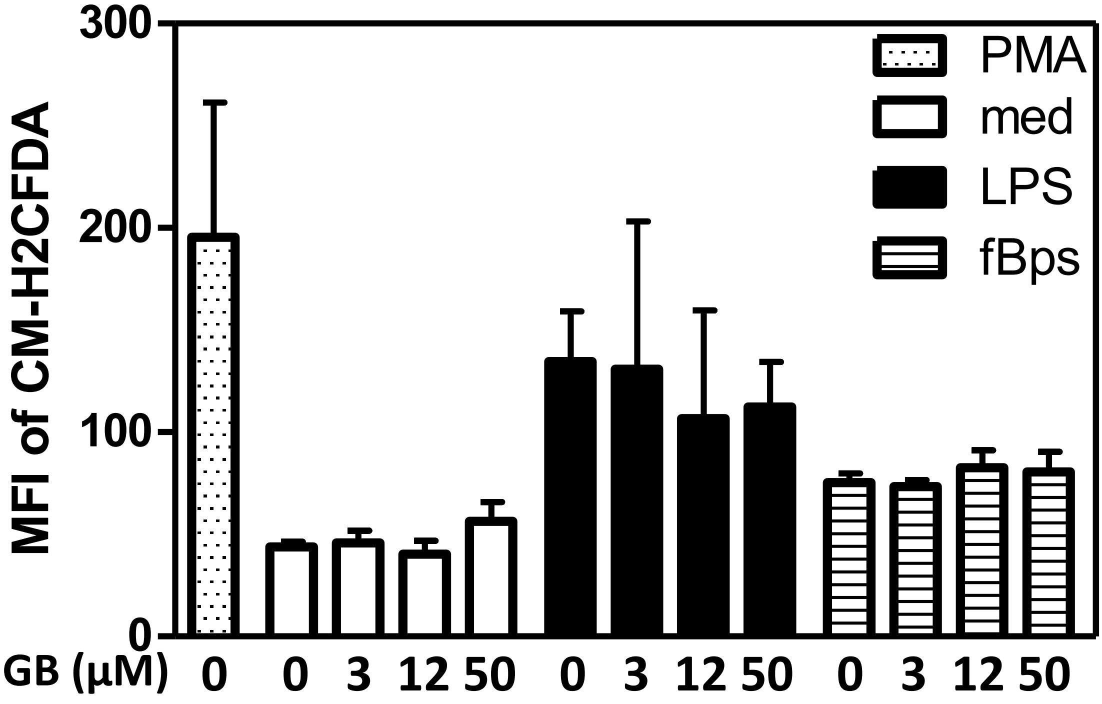

Supplementary Figure S1: Glibenclamide does not affect reactive oxygen species (ROS) production by PMNs. Purified PMNs from healthy individuals were treated with glibenclamide (GB, 3, 12 and 50 µM) for 1 h. After treatment PMNs were incubated with 1 µg/ml LPS or fixed *B. pseudomallei* killed by 1% paraformaldehyde (ratio 1:10) for 1 h and then the PMNs were collected for detection of CM-H2CFDA (General oxidative stress indicator) by flow cytometry. Statistical analysis was performed using Two Way ANOVA comparing each bar of GSH treatment group with no GB of each GB treatment group. The results are expressed as mean ± s.d. from 1 of 2 independent experiments. No asterisk, non significant.

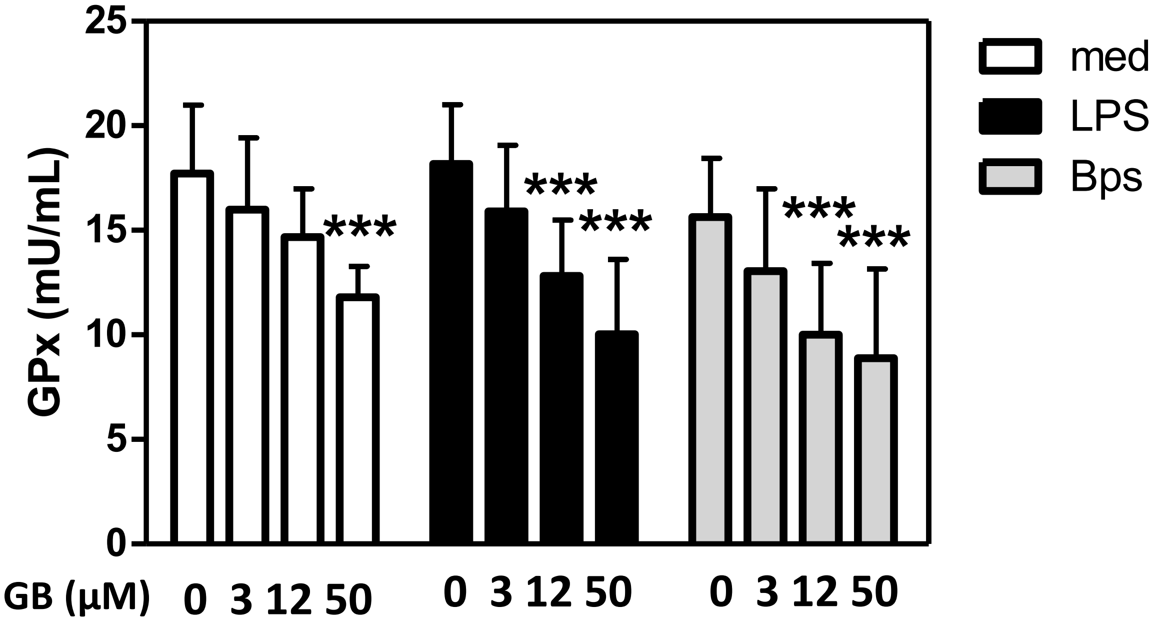

Supplementary Figure S2: Glibenclamide inhibits glutathione peroxidase (GPx) activity of PMNs. Purified PMNs from healthy individuals were treated with glibenclamide (GB, 3, 12 and 50 µM) for 1 h. After treatment PMNs were incubated with 1 µg/ml LPS or live *B. pseudomallei* (MOI 1:1) for 1 h and then the PMNs were collected for detection of glutathione peroxidase (GPx) activity. Statistical analysis was performed using Two Way ANOVA comparing each bar of GSH treatment group with no GB of each GB treatment group. The results are expressed by mean ± s.d. from 3 independent experiments, and samples were assayed in duplicated. ****P<0.001.* No asterisk, non significant.

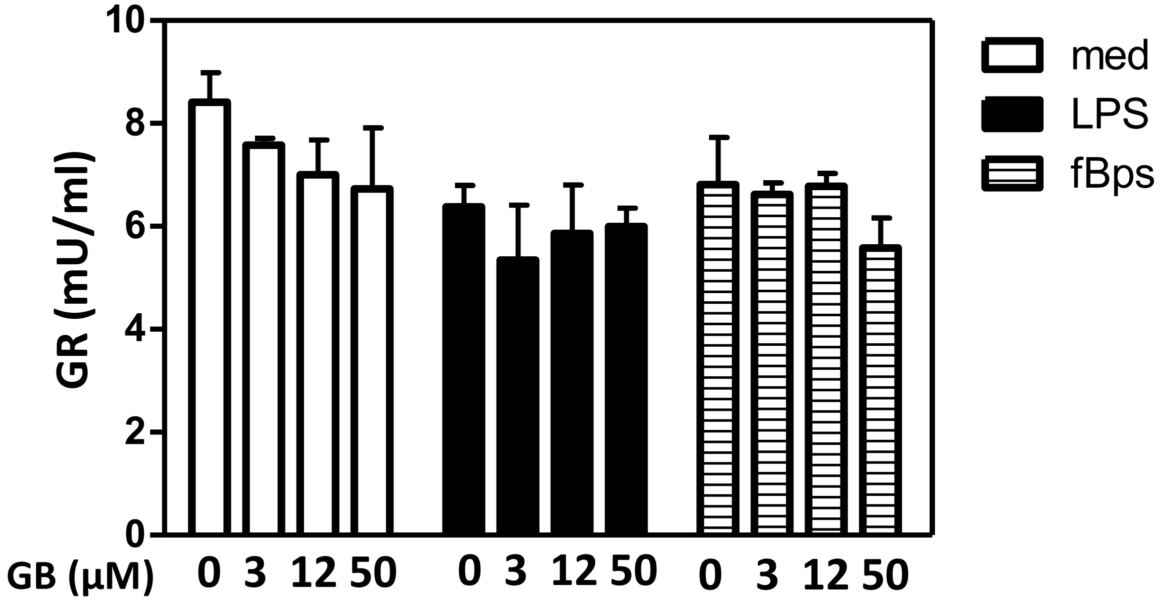

Supplementary Figure S3: Glibenclamide does not affect glutathione reductase (GR) activity of PMNs. Purified PMNs from healthy individuals were treated with glibenclamide (GB, 3, 12 and 50 µM) for 1 h. After treatment PMNs were incubated with 1 µg/ml LPS or fixed *B. pseudomallei* (MOI 10) for 1 h and then the PMNs were collected for detection of glutathione reductase (GR) activity. Statistical analysis was performed using Two Way ANOVA comparing each bar of GSH treatment group with no GB of each GB treatment group. The results are expressed as mean ± s.d. from 2 independent experiments. No asterisk, non significant.
